# Plausible Mechanism of Sham Acupuncture Based on Biomarkers: A Systematic Review of Randomized Controlled Trials

**DOI:** 10.3389/fnins.2022.834112

**Published:** 2022-02-03

**Authors:** Tae-Hun Kim, Myeong Soo Lee, Stephen Birch, Terje Alraek

**Affiliations:** ^1^Korean Medicine Clinical Trial Center, Korean Medicine Hospital, Kyung Hee University, Seoul, South Korea; ^2^KM Science Research Division, Korea Institute of Oriental Medicine, Daejeon, South Korea; ^3^School of Health Sciences, Kristiania University College, Oslo, Norway; ^4^Department of Community Medicine, Faculty of Medicine, National Research Center in Complementary and Alternative Medicine, UiT the Arctic University of Norway, Tromso, Norway

**Keywords:** biomarkers, acupuncture mechanism, sham acupuncture, systematic review, meta-analysis

## Abstract

**Introduction:**

Sham acupuncture was developed to be used as an inert control intervention in clinical trials of acupuncture. However, controversies exist regarding the validity of sham acupuncture. In this systematic review (SR) of acupuncture trials, we assessed whether serum biomarkers showed significant differences after sham and verum acupuncture treatments.

**Methods:**

Any acupuncture clinical trials that evaluated serum biomarker changes between sham acupuncture and verum acupuncture were included in this review. Relevant literature was searched in the PubMed database, EMBASE, and The Cochrane Central Register of Controlled Trials (CENTRAL) database from inception until June 2021. The Cochrane risk of bias was assessed. Summary effect estimates for each biomarker between groups were calculated with a random effect model.

**Results:**

From 51 sham acupuncture trials, we found that there were no significant differences in most of the 36 serum biomarkers after sham acupuncture and verum acupuncture needling. Only VEGF, IG-E, TNF-a, NGF, GABA, NPY, and VIP serum levels were identified as being different between the groups. The overall risk of bias of the included studies and the limited numbers of studies for meta-analysis do not strongly support the results of this SR.

**Conclusion:**

Sham acupuncture techniques might have similar effects on biomarkers as the so-called “real acupuncture” techniques, which indicates that sham acupuncture, as an inert intervention similar to a placebo drug, needs to be reconsidered.

**Systematic Review PROSPERO Registration:**

identifier [CRD42021260889].

## Introduction

Sham acupuncture was developed to be used as an inert control intervention in clinical trials of acupuncture. It was supposed to be similar to placebo pills in pharmacological studies or inert-sham treatments in technique or device studies (Birch et al., 2021). Therefore, the impression people/researchers usually have about sham acupuncture is that it is (or should be) an intervention that is essentially identical to acupuncture in external appearance but has no physiological effects. If this idea is correct, sham acupuncture should not exert any specific effects of acupuncture other than the expected nonspecific effects of a placebo.

However, there have been controversies around the validity of sham acupuncture as an appropriate control intervention in randomized placebo-controlled acupuncture trials (Lund and Lundeberg, 2006). Although at least blinding of the patients is achievable, acupuncture practitioners are inevitably aware of which acupuncture method they apply, which means that perfect prevention of performance bias is not possible (Trinh, 2003). The situation becomes more complex when we examine the question of “what is acupuncture” and what the associated physiological effects of the different types of needling are. Sham techniques were developed before a mature body of literature about what constitutes the practice of acupuncture had emerged (Birch et al., 2021). What was tested in many clinical trials (so-called “real acupuncture”) was usually one type of needling method, while other treatment techniques, seeking to avoid the sensory stimulation of that method, that may have accidentally mimicked other styles of acupuncture were used as so-called “sham acupuncture” (Wei-Xing, 2019). Empirical evidence suggests that so-called “sham acupuncture” might have physiological effects, not surprising given the confusion about what constitutes the practice of acupuncture (Wei-Xing, 2019) and predictable physiological effects of different forms of tissue stimulation (Zhang et al., 2012). These issues have sparked debate about the inadequacy of currently used sham acupuncture as a proper control intervention (Zhang et al., 2012; Birch et al., 2022). Although many researchers talk about the comparison of “real” and “sham” acupuncture in their clinical trials, we do not accept this comparison as being accurate, and the term “real” is highly misleading and often inaccurate; instead, we adopt the use of “verum” vs. “sham” acupuncture, as it more accurately captures the assumptions that different research groups have made.

Scientific evidence is needed to evaluate whether what has been called “sham acupuncture” is an appropriate choice in acupuncture studies. If sham acupuncture does not have any physiological effects beyond placebo, it would be reasonable to expect that the various physiological changes in the human body caused by a verum acupuncture treatment, such as changes in neurotransmission or brain activity, as well as changes in endocrinologic secretion, do not appear. Against this background, as a first step, we examined whether there is a difference in the expression of serum biomarkers between verum and sham acupuncture treatments included in acupuncture clinical trials. If there was an apparent difference in the physiological effects of sham acupuncture and verum acupuncture methods, there would be a different presentation of principle biomarkers secreted in the serum after application of the two types of acupuncture. Our primary hypothesis of this review was that sham acupuncture may influence biomarkers, similar to verum acupuncture. In addition, a secondary hypothesis was that the biomarker effect of sham acupuncture may offer potential explanations for how “sham techniques” can influence health problems such as pain and inflammation.

## Methods

To evaluate sham acupuncture-related serum biomarker changes compared with those of verum acupuncture, we conducted a systematic review (SR) of acupuncture trials that compared serum biomarkers between sham and verum acupuncture groups regardless of health status and acupuncture type. The protocol of this review was registered in PROSPERO (CRD42021260889). This study was conducted following the guidance of PRISMA 2020 statements (Supplementary File 1).

### Eligibility Criteria

In this SR, we included any randomized controlled trials (RCTs) comparing serum biomarker secretion between sham and verum acupuncture methods. The following PICO components were considered when evaluating eligibility criteria:

Population: Both healthy participants and patients with any type of disease were included in this study.

Intervention: Any type of sham acupuncture used in clinical trials, including skin stimulation without acupuncture needles, such as cocktail sticks or toothpicks, superficial needling on acupuncture points or nonacupuncture points, and sham devices without penetration (Park, Streitberger, and Takakura sham needles), was included in this review.

Comparator: Any types of verum acupuncture regardless of acupuncture points, needle types, stimulation methods (manual or electroacupuncture), duration and frequency, and combination therapies were included in this review as long as the study authors described their interventions as a verum acupuncture in the study.

Outcome: Serum biomarkers, including cytokines, serotonin, acetylcholine, AMP, AMPK, ATP, BDNF, Ca2+, cAMP, CGRP, chemokines, DNA, eNOS, erythropoietin, FGF-2, GABA, H+, interleukin, K+, norepinephrine, NGF, NO, PG, TGF, TNF, irisin, leukemia inhibitory factor, opioid, neuropeptide, prostaglandin, and myokines, needed to be assessed in the included studies. We used values at the time point of primary outcome measurement after acupuncture treatment in each study. If there were only change scores available between before-and-after treatments, we extracted change scores instead.

Types of studies: In this review, we only included RCTs comparing serum biomarkers between sham acupuncture and verum acupuncture. Crossover trials were excluded.

### Information Sources and Searching Strategy

Relevant literature was located through core electronic database searching, including the PubMed database, EMBASE, and The Cochrane Central Register of Controlled Trials (CENTRAL) database, from the inception until June 2021. We did not impose any language restrictions. Medline database searching was conducted through PubMed with the following search strategy:

#1 acupuncture
#2 sham acupuncture
#3 biomarker OR cytokine OR serotonin OR acetylcholine OR AMP OR AMPK OR ATP OR BDNF OR Ca OR cAMP OR CGRP OR Chemokines OR DNA OR eNOS OR erythropoietin OR FGF-2 OR GABA OR Interleukin OR norepinephrine OR NGF OR PG OR TGF-B OR TNF OR Irisin OR leukemia inhibitory factor OR opioid OR neuropeptide OR Prostaglandin OR myokines
#4 (#1 OR #2) AND #3 Filters: Clinical Trial.

### Selection Process and Data Extraction

Two review authors (T-HK and SB) conducted study selection individually and discussed the results. The third arbiter (MSL) decided to include or exclude studies if there were conflicts between the two authors. Study characteristics, including the number of participants, conditions, type of sham acupuncture and verum acupuncture, duration of interventions, time of the outcome assessments and detailed values of each serum biomarker after treatments in both the sham and verum acupuncture groups, were extracted by two authors (T-HK and SB) individually, and the results were discussed between the authors. In general, various serum biomarkers were evaluated at multiple time points in a study. The first outcome immediately after the end of treatment was selected for analysis in this review. Apart from the serum concentrations of biomarkers, to collect data on the differences between groups that the authors judged narratively, sentences that mentioned biomarkers in the discussion or conclusion sections were collected.

### Quality Assessment

The Cochrane risk of bias (ROB 1) tool was used to assess the quality of the included studies. Six domains, namely, sequence generation, allocation concealment, blinding of participants and outcome assessors, incomplete outcome data, selective reporting, and other bias domains, were assessed. If any of the domains had a high risk of bias or unclear bias, the overall risk of bias was decided to be a high risk of bias. Only a low risk of bias for overall domains was suggested when there was no high or unclear domain in each study.

### Synthesis

Because the purpose of our review was to examine whether there were significant differences in the secretion of serum biomarkers between the sham and verum acupuncture groups, a meta-analysis was conducted for each biomarker comparing sham acupuncture with verum acupuncture regardless of the type of intervention and participant condition. Although, it is difficult to classify biomarkers because they are involved in complex mechanisms, to help with a general understanding, we divided biomarkers into the following categories: those related to immunity and inflammation (inflammation and immune-modulatory biomarkers), those related to metabolism (metabolic biomarkers) and those related to pain (neuromodulatory biomarkers). We only provided summary effect estimates for each biomarker; however, a meta-analysis of all studies was not conducted because it was judged to be inappropriate to synthesize overall effect estimates of sham acupuncture for the secretion of different types of biomarkers. The random effect model was used due to potential clinical heterogeneity of the included studies. If the included studies presented the quantity of biomarker secretion in different units, we used generic inverse variance meta-analysis to calculate the standard mean difference (SMD) with 95% confidence intervals for each biomarker. For the interpretation of the meta-analysis result, we used a rule of thumb criteria such as 0.3 of SMD for a small difference, 0.6 for a moderate difference, and 0.9 for a large difference. Meta-analysis for individual biomarker secretion was conducted using the metagen package in R software (Ver. 4.1.1), and a forest plot was presented using the RevMan program (Ver 5.3). Subgroup analysis based on the subtype of sham acupuncture and acupuncture stimulation methods (manual acupuncture and electroacupuncture) was intended to be done, but only a small number of studies evaluating the same biomarkers were included in this review; hence subgroup analysis was not conducted. Publication bias was assessed with visual inspection of funnel plot.

## Results

### Summary of the Included Studies

**Figure 1:**
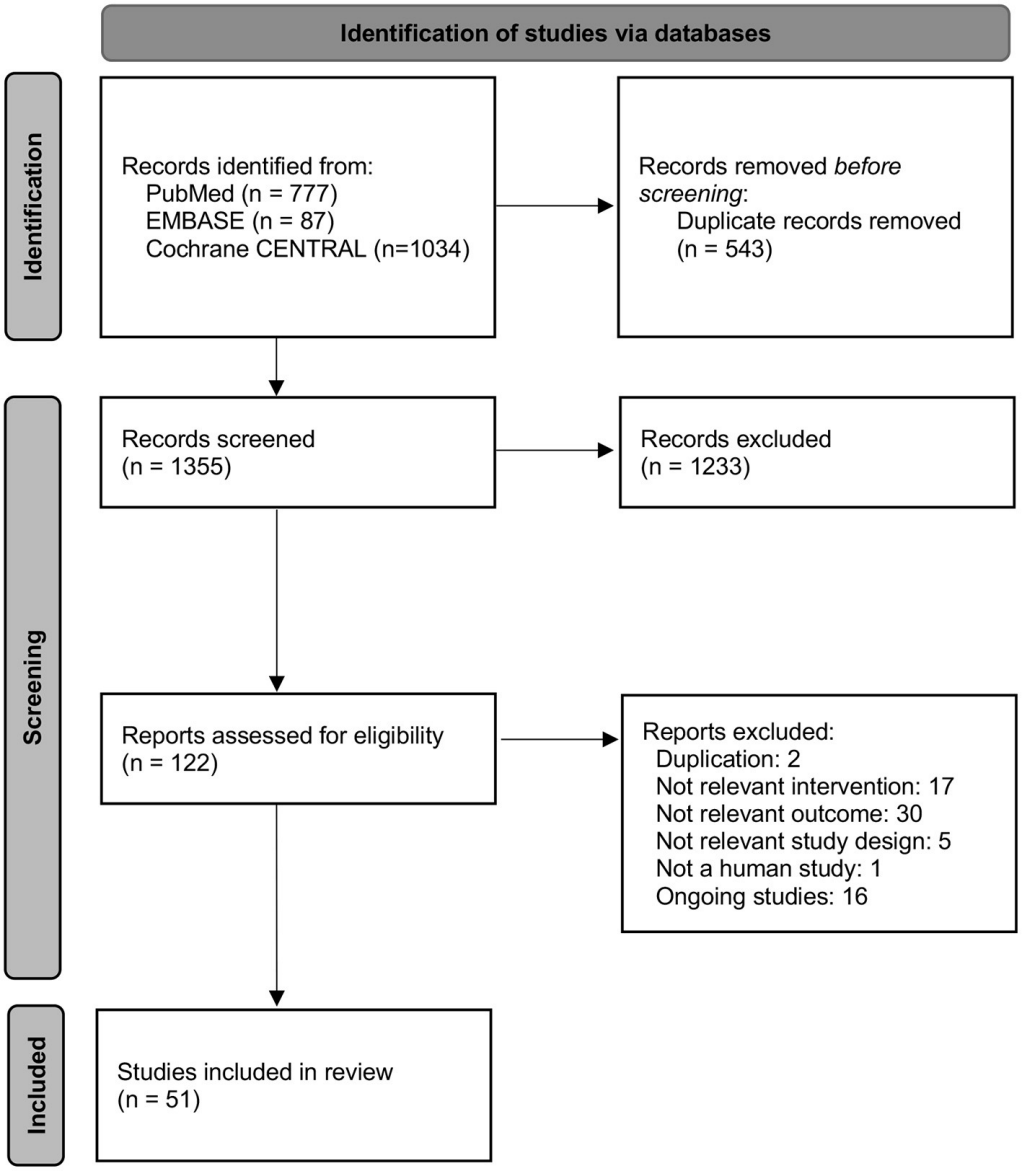
Flow diagram.

Among 1898 studies located through electronic database searching, 51 sham acupuncture trials were included in this review (Figure 1). Studies included different patients or populations, specifically healthy adults (*n* = 7) or those with musculoskeletal disease (*n* = 6), gastrointestinal disease (*n* = 5), mental disease (*n* = 4), cancer (*n* = 4), obesity (*n* = 4), cardiovascular disease (*n* = 3), neurologic disease (*n* = 3), gynecologic disease (*n* = 3), genitourinary disease (*n* = 2), sexually transmitted disease (*n* = 2), and miscellaneous (*n* = 8). Sham acupuncture types were skin stimulation with acupuncture needles or without acupuncture needles such as cocktail sticks or toothpicks (*n* = 13), superficial needling on acupuncture points or nonacupuncture points (*n* = 24), and sham devices without penetration (the Park, Streitberger and Takakura sham devices) (*n* = 14). The intervention period showed vast heterogeneity among the included studies, from 1 day of treatment to 16 weeks of treatment (Supplementary Files 2, 3). Twenty-one studies showed a low risk of bias, and 30 studies showed a high risk of bias. Domains such as sequence generation, allocation concealment and blinding of outcome assessors were unclear in the high risk of bias studies (Supplementary File 4).

### Inflammation and Immune-Modulatory Biomarkers

**Figure 2:**
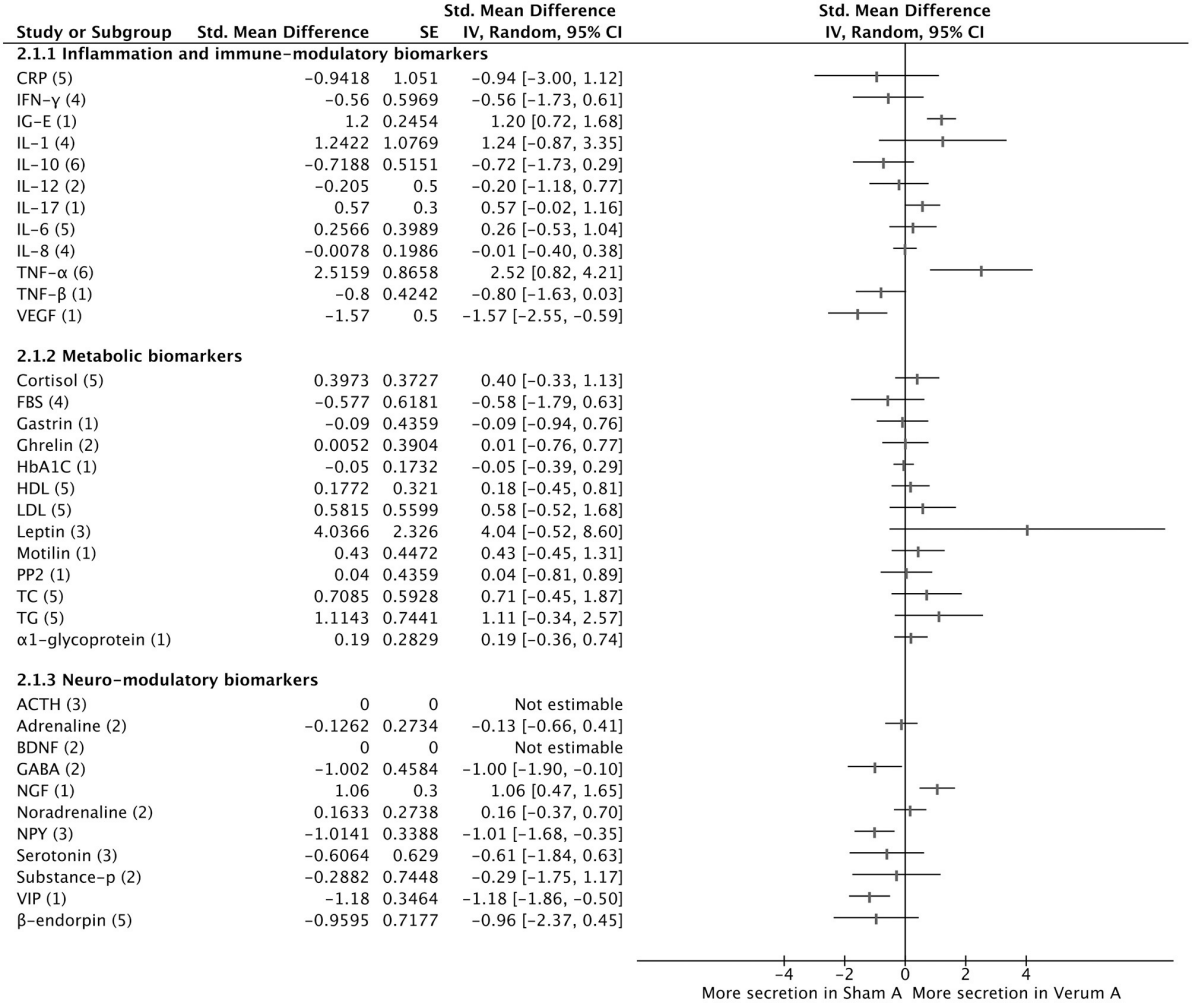
Comparison of serum biomarkers secretion between sham acupuncture group and test acupuncture group. The number in parentheses indicates the number of studies included in the meta-analysis. BDNF, brain derived neurotrophic factor; CI, Confidence intervals; CRP, C-reactive protein; FBS, fasting blood glucose; GABA, γ-aminobutyric acid; HDL, high-density lipoprotein cholesterol; IFN-γ, Interferon gamma; IL, interleukin; LDL, low-density lipoprotein cholesterol; NGF, nerve growth factor; NPY, Neuropeptide Y; NR, not reported; PP2, 2-h postprandial blood glucose; SP, substance-P; Test A: TC, total cholesterol; TG, Triglycerides; TNF-α, tumor necrosis factor-α; VIP, vasoactive intestinal peptide; Sham A, Sham acupuncture; Verum A, Verum acupuncture.

Among the evaluated inflammation and immune-modulatory biomarkers, the sham acupuncture groups showed a significant decrease in serum VEGF levels (−1.57, 95% CI [−2.55 to −0.59]) and serum IG-E levels (1.20, 95% CI [0.72 to 1.68]) and a significant decrease in serum TNF-α levels (2.52, 95% CI [0.82 to 4.21]) compared with those of the verum acupuncture groups. However, there were no significant differences between the two sham acupuncture and verum acupuncture groups in the other biomarkers (CRP, IFN-c, IFN-γ, IL-1, IL-10, IL-12, IL-6, and TNF- β) (Figure 2).

### Metabolic Biomarkers

None of the metabolic biomarkers showed significant differences between the sham and verum acupuncture groups (Figure 2).

### Neuromodulatory Biomarkers

Among the evaluated neuromodulatory biomarkers, there were significant differences in the serum GABA levels (−1.00, 95% CI [−1.90, −0.10]), NGF levels (1.06, 95% CI [0.47 to 1.65]), NPY levels (−1.01, 95% CI [−1.68, −0.35]) and VIP levels (−1.18, 95% CI [−1.86, −0.50]), which suggested that the verum acupuncture groups showed significantly more secretion of serum GABA, NPY and VIP than the sham acupuncture groups. However, serum adrenaline, B-endorphin, HT-5 and noradrenaline did not show differences between the sham acupuncture and verum acupuncture groups.

### Publication Bias

From the visual inspection of funnel plot, most studies were scattered symmetrically in a triangle centered on 0. However, this result could not ensure that there was no publication bias in this study because only small numbers of studies were included in the meta-analysis (Supplementary File 5).

## Discussion

From 51 sham acupuncture trials, we found that most of the 36 serum biomarkers did not suggest a significant difference between sham acupuncture and verum acupuncture needling. Among the evaluated inflammation and immune-modulatory biomarkers, VEGF, IG-E, and TNF-a levels were identified to have group differences. NGF among the metabolic biomarkers and GABA, NPY, and VIP serum levels were identified to be different between groups. Due to the overall risk of bias of the included studies and the limited number of studies for meta-analysis not strongly supporting the results of this SR, caution is required in the interpretation of the findings of this study.

Sham acupuncture, if it follows the originally intended definition, should not have any of the active physiological effects that verum or so-called “real acupuncture” has (Thomas and Fitter, 2002). However, sham acupuncture techniques were not developed appropriately with regard to elimination or control of potential physiological effects (Birch et al., 2021), nor did the development of sham techniques take into account the extensive physiological studies that have been conducted or the wide variety of acupuncture practices worldwide. Sham acupuncture has been understood by many researchers to be a mere dummy intervention, with the intention of evaluating the efficacy of *real* acupuncture, without consideration of whether sham acupuncture techniques have physiological effects beyond placebo. As a consequence, there have been many studies that have drawn the premature conclusion, intended or not, that acupuncture treatment was only a placebo for many diseases because it was not significantly more effective than sham (So et al., 2009; Fregni et al., 2010), rather than acknowledging the potential type II errors due to insufficient sample sizes (Lewith and Machin, 1983) and potentially biasing against acupuncture (Birch, 2006; Appleyard et al., 2014; MacPherson et al., 2014). From this brief review, we found that there were no significant differences between the sham and verum acupuncture groups in the level of most serum biomarkers, which indirectly means that sham acupuncture techniques can also show the same physiological changes as verum acupuncture techniques. We can predict that similar physiological effects will occur with both the sham and verum acupuncture needling techniques since more superficial stimulation will influence the same receptors regardless of shallower or deeper stimulation techniques (Zhang et al., 2012), independent of placebo. Zhang and colleagues also found that the Streitberger, Park, and Takakura nonpenetrating sham devices are clinically effective beyond placebo effects (Zhang et al., 2015). The results of the present study provide further evidence for the need to reconsider whether so-called sham acupuncture is an appropriate type of control intervention in acupuncture trials.

One area that needs discussion are concerns regarding the differential mechanisms of sham acupuncture and verum acupuncture techniques. Among the inflammation and immune-modulatory biomarkers, the serum levels of TNF-α and IG-E were higher in the verum acupuncture groups than in the sham acupuncture groups. For neuromodulatory biomarkers, GABA, NGF, NPY, and VIP were found to be higher in the verum acupuncture groups than in the sham acupuncture groups. We failed to identify any trends in the changes in these biomarkers when comparing the effects of sham and verum acupuncture methods. Whether this difference is derived from a real difference in the mechanism of action of the two types of acupuncture, or whether it was derived by chance due to the small number of included studies for meta-analysis needs to be clarified through a comparative study on acupuncture and sham acupuncture experimental studies in the future.

This study has limitations. First, a clinical heterogeneity issue could exist in the meta-analyses of this study. We included studies for analysis if the study used acupuncture and sham acupuncture and evaluated biomarkers regardless of disease type, acupuncture, and sham acupuncture modalities (or points), stimulation duration, frequency, and severity. Since there are debates about how acupuncture can act bidirectionally depending on the disease, patient condition or stimulation methods, this clinical heterogeneity is important in interpreting the results of our study (Wei-Xing, 2019). Comparative studies under strictly controlled conditions, such as in a laboratory, are needed. Second, chemical biomarkers appearing in serum were used in this study, and there may be a problem in classifying them into three types. Biomarkers are defined as measurable indicators for assessing the human condition or diagnosing diseases in medicine (Strimbu and Tavel, 2010). Vital signs, including pulse and blood pressure, can be included as examples of biomarkers. Substances that are recognized as biomarkers are difficult to classify into one category because they have various actions depending on the site itself. In this study, the classification of biomarkers into three types was carried out for the convenience of understanding. Third, we could not evaluate differential effects induced by the acupuncture stimulation methods (manual acupuncture or electroacupuncture) or type of sham acupuncture due to limited numbers of included studies for each biomarker. Fourth, we did not include searching for regional databases including Chinese databases and Korean databases in addition to clinical trial registries. In addition to this, there could be missing studies which might be introduced from our searching strategy. Fifth, we could not assess all the potential biomarkers which might be related to the differential effects of sham acupuncture and real acupuncture. We only assessed 36 serum biomarkers which had been assessed in clinical trials and there could be another type of biomarkers which might play important roles during the acupuncture stimulation. From this point of view, publication bias might be introduced to the study results. These limitations should be considered when interpreting the result of this study.

In conclusion, sham acupuncture techniques might have similar effects on biomarkers that so-called “real acupuncture” techniques have. Acupuncture clinical studies conducted under the assumption that sham acupuncture is an inert intervention much like a placebo drug need to be reconsidered.

## Data Availability Statement

The original contributions presented in the study are included in the article/Supplementary Material, further inquiries can be directed to the corresponding author/s.

## Author Contributions

T-HK, SB, TA, and ML: conceptualization, methodology, writing—original draft, writing—review, editing, and visualization. T-HK and ML: resources. All authors contributed to the article and approved the submitted version.

## Funding

This study was funded by Korea Institute of Oriental Medicine (KSN2021210).

## Conflict of Interest

The authors declare that the research was conducted in the absence of any commercial or financial relationships that could be construed as a potential conflict of interest.

## Publisher's Note

All claims expressed in this article are solely those of the authors and do not necessarily represent those of their affiliated organizations, or those of the publisher, the editors and the reviewers. Any product that may be evaluated in this article, or claim that may be made by its manufacturer, is not guaranteed or endorsed by the publisher.

## Supplementary Material

Supplementary File 1 PRISMA checklist 2020.

Supplementary File 2 Summary of the included studies.

Supplementary File 3 References of the included studies.

Supplementary File 4 Risk of bias assessment.

Supplementary File 5 Funnel plot.

## References

[B1] Appleyard I. Lundeberg T. Robinson N. (2014). Should systematic reviews assess the risk of bias from sham-placebo acupuncture control procedures? Eur. J. Integr. Med. 6, 234–243. 10.1016/j.eujim.2014.03.00422592702

[B2] Birch S. (2006). A review and analysis of placebo treatments, placebo effects, and placebo controls in trials of medical procedures when sham is not inert. J. Altern. Complem. Med. 12, 303–310. 10.1089/acm.2006.12.303 16646730

[B3] Birch S. Lee M. S. Kim T.-H. Alraek T. (2021). Historical perspectives on using sham acupuncture in acupuncture clinical trials. Integr. Med. Res. 11:100725. 10.1016/j.imr.2021.100725 34458094 PMC8379290

[B4] Birch S. Lee M. S. Kim T.-H. Alraek T. (2022). On defining acupuncture and its techniques: A commentary on the problem of sham. Integr. Med. Res. 100834. 10.1016/j.imr.2022.100834 35111572 PMC8790499

[B5] Fregni F. Imamura M. Chien H. F. Lew H. L. Boggio P. Kaptchuk T. J. . (2010). Challenges and recommendations for placebo controls in randomized trials in physical and rehabilitation medicine: a report of the international placebo symposium working group. Am. J. Phys. Med. Rehabil. 89:160. 10.1097/PHM.0b013e3181bc0bbd 20090428 PMC2900312

[B6] Lewith G. T. Machin D. (1983). On the evaluation of the clinical effects of acupuncture. Pain 16, 111–127. 10.1016/0304-3959(83)90202-6 6348651

[B7] Lund I. Lundeberg T. (2006). Are minimal, superficial or sham acupuncture procedures acceptable as inert placebo controls? Acupunct. Med. 24, 13–15. 10.1136/aim.24.1.13 16618044

[B8] MacPherson H. Vertosick E. Lewith G. Linde K. Sherman K. J. Witt C. M. . (2014). Influence of control group on effect size in trials of acupuncture for chronic pain: a secondary analysis of an individual patient data meta-analysis. PLoS One 9:e93739. 10.1371/journal.pone.0093739 24705624 PMC3976298

[B9] So E. W. S. Ng E. H. Y. Wong Y. Y. Lau E. Y. L. Yeung W. S. B. Ho P. C. (2009). A randomized double blind comparison of real and placebo acupuncture in IVF treatment. Hum. Reprod. 24, 341–348. 10.1093/humrep/den380 18940896

[B10] Strimbu K. Tavel J. A. (2010). What are biomarkers? Curr. Opin. HIV AIDS 5:463. 10.1097/COH.0b013e32833ed177 20978388 PMC3078627

[B11] Thomas K. Fitter M. (2002). Possible research strategies for evaluating CAM interventions, in Clinical Research in Complementary Therapies (Elsevier), 59–91.

[B12] Trinh K. V. (2003). Blinding in acupuncture research: a systematic review of randomized controlled trials for pain using a sham acupuncture control. Clin. Acupunct. Orient. Med. 4, 71–77. 10.1016/S1461-1449(03)00026-4

[B13] Wei-Xing P. (2019). Bidirectional regulation of acupuncture and its plausible mechanisms. Acupunct. Res. 11, 843–853. 10.13702/j.1000-0607.190260 31777237

[B14] Zhang C. S. Tan H. Y. Zhang G. S. Zhang A. L. Xue C. C. Xie Y. M. (2015). Placebo devices as effective control methods in acupuncture clinical trials: a systematic review. PLoS One 10:e0140825. 10.1371/journal.pone.0140825 26536619 PMC4633221

[B15] Zhang Z.-J. Wang X.-M. Mcalonan G. M. (2012). Neural acupuncture unit: a new concept for interpreting effects and mechanisms of acupuncture. Evid. Based Complem. Altern. Med. 2012:429412. 10.1155/2012/429412 22474503 PMC3310280

